# Secular trends of bloodstream infections in hemodialysis patients: insights from a longitudinal Swiss study

**DOI:** 10.1186/s13756-025-01620-8

**Published:** 2025-08-20

**Authors:** Nasreen Hassoun-Kheir, Niccolò Buetti, David Jaques, Valérie Olivier, Marie-Noelle Chraiti, Monique Perez, Marlieke EA de Kraker, Holly Jackson, Jacques Schrenzel, Patrick Saudan, Stephan Harbarth

**Affiliations:** 1 Infection Control Program, Faculty of Medicine, Geneva University Hospitals, WHO Collaborating Center, Geneva, Switzerland; 2 INSERM, IAME, Université Paris-Cité, Paris, France; 3 Nephrology division, Faculty of Medicine, Geneva University Hospitals, Geneva, Switzerland; 4 Bacteriology Laboratory, Division of Laboratory Medicine, Geneva, Switzerland; 5 Genomic Research Laboratory, Division of Infectious Diseases, Geneva University Hospitals and Faculty of Medicine, Geneva, Switzerland

**Keywords:** Bloodstream infections, Hemodialysis, Epidemiological trends

## Abstract

**Background:**

Hemodialysis-associated bloodstream infections (BSIs) represent a significant burden for patients. Understanding the trends in BSIs among hemodialysis patients is crucial for informing strategies to reduce their incidence and improve patient outcomes. This study aimed to evaluate secular trends, identify causative organisms, assess resistance patterns, and determine the sources of hemodialysis-associated BSIs at Geneva University Hospitals, where *Staphylococcus aureus* screening and decolonization of hemodialysis patients have been implemented since the year 2000.

**Methods:**

A longitudinal cohort study was conducted using data from 2006 to 23. We included all patients receiving maintenance hemodialysis treatment at our institution. A hemodialysis-associated BSI was defined as BSI occurring during active hemodialysis treatment and diagnosed either during hospital admission or in outpatient hemodialysis unit. Outcomes included incidence rates of hemodialysis-associated BSIs, trends in causative pathogens, sources, and resistant organisms. Poisson regression was used to model trends over time of incidence rate ratios (IRR).

**Results:**

A total of 313 true BSI episodes were identified in 218 hemodialysis patients over 11,413 patient-hemodialysis months. The overall BSI incidence rate was 2.7 episodes per 100 patient-hemodialysis-months, with a consistent decrease over time. Compared to 2006-08, hemodialysis-associated BSI rates decreased by 16% in 2009-11 (IRR 0.84, 95% confidence interval [CI] 0.60–1.18), and by a maximum of 44% in 2021-23 (IRR 0.56, 95% CI 0.36–0.83). The decreasing trend was mainly due to reduced *S. aureus* BSIs, while Enterobacterales BSIs rates remained stable. Catheter-related BSIs accounted for 41.5% of infections (130/313), with marked reduction following 2014. BSIs caused by resistant bacteria were rare, with decreasing trends of methicillin-resistant *S. aureus*.

**Conclusions:**

Hemodialysis-associated BSI rates significantly declined, driven largely by reductions in *S. aureus* BSIs and catheter-related infections. No replacement by Gram-negative BSI was observed. Prevention of hemodialysis-associated BSI is key for reducing infection burden among hemodialysis patients.

**Supplementary Information:**

The online version contains supplementary material available at 10.1186/s13756-025-01620-8.

## Background

Infections are a leading cause of mortality in patients undergoing maintenance hemodialysis (HD), with an infection-related death rate of 18.5 per 1000 person-years, which is approximately 20 times higher than that of the general population [1]. BSI fatality rates globally range between 18 and 30%, and they often require hospitalization [2–4]. HD patients are at increased risk of infections due to several factors including comprised immunity, need for long-term vascular access and frequent contact with the healthcare system [5, 6]. Bloodstream infections (BSIs) are particularly prevalent, often complicating regular hemodialysis treatments and imposing a significant burden on these patients [7, 8]. A population-based cohort study found that HD patients had a 26-fold higher risk of BSI compared to an age- and gender-matched control group [3]. Common causative pathogens for BSI include *Staphylococcus aureus*, coagulase-negative staphylococci (CONS), *Klebsiella pneumoniae*,* Escherichia coli* and *Pseudomonas aeruginosa* [2]. BSI burden is exacerbated by the high risk for colonization with multidrug resistant organisms (MDROs) and related difficult-to-treat infections [7, 8].

The risk for developing BSI is highly influenced by the type of the HD-vascular access, with central venous catheters (CVCs) carrying the highest risk [7]. Data from the US hemodialysis surveillance report (2014-19), indicate that 63% of BSIs occurred in patients with CVCs, despite CVCs representing only 20% of the total surveyed patient months [9]. This finding underscores the critical importance of implementing effective infection control measures and proper catheter care to mitigate this risk of BSIs in such a vulnerable patient population. There is a large potential for BSI prevention in HD patients; a previous study conducted across 17 U.S. outpatient hemodialysis units showed that implementing a comprehensive infection control protocol resulted in a 32% reduction in overall BSI rates and a 54% decrease in access-related BSIs [10]. Among the infection control activities at our institution, a targeted intervention specifically addressing *S. aureus* in HD patients was initiated in the year 2000, involving a bimonthly screening for *S. aureus* carriage and subsequent decolonization of identified carriers [11].

The incidence and microbial etiology of BSIs in HD patients differs from that of the general population [12]. To date , only few studies have reported the secular trends and changes in causative pathogens and antimicrobial resistance patterns in bacteremic patients undergoing HD [10, 12, 13]. In this study, we aimed to describe and quantify trends, causative pathogens, and resistance patterns of healthcare-related BSIs among patients receiving maintenance HD in our institution. We further sought to explore whether Gram-negative bacteria have replaced *S. aureus* as causative pathogens in HD patients following the implementation of *S. aureus* targeted screening and decolonization. We hypothesized that while the rates of methicillin-sensitive and methicillin-resistant *S. aureus* (MSSA and MRSA) BSIs decreased with time, the incidence of BSIs caused by Gram-negative pathogens remained stable.

## Methods

### Study setting and population

We conducted a longitudinal cohort study using prospectively collected data on healthcare-related BSI between 1 January 2006 and 31 December 2023 at Geneva University Hospitals (HUG), a 2100-bed university-affiliated tertiary care hospital in Geneva, Switzerland. The HD unit at our institution has the capacity of offering maintenance dialysis for approximately 60 patients. Prospective surveillance of healthcare-related BSI is performed routinely by the infection prevention and control team, including all nosocomial BSI episodes occurring > 48 h after hospital admission or within 30 days of receiving ambulatory care, including those identified in blood cultures collected in outpatient clinics [14]. Patients receiving maintenance HD in the institution were identified through a dedicated HD patient registry [15]. HD-associated BSI episodes included episodes detected during hospitalization of HD patients or HD outpatient clinic visits. Patients receiving peritoneal dialysis were excluded. Patients undergoing intermittent HD contributed to the analysis only during corresponding active HD-periods.

### Data collection and definitions

Standardized BSI surveillance reporting was implemented at our institution in 2006. Data were collected from BSI surveillance reports and a dedicated HD patient registry. A BSI episode was defined as isolation of bacteria or fungi from ≥ 1 blood culture in a HD patient during a period of ongoing HD. BSI episodes were classified as monomicrobial or polymicrobial. BSI episodes were also classified as primary/secondary BSIs, and contamination episodes were excluded. Skin commensals were considered contaminants unless they appeared in at least two blood culture sets that were collected at different times alongside clinical signs of infection [16]. BSI classification was done according to the Centers for Disease Control and Prevention (CDC) guidelines during the years 2006-15, and the European Centre for Disease Prevention and Control (ECDC) guidelines from 2016 onwards [17]. In summary, primary BSIs originate directly in the bloodstream without an identifiable source, while secondary BSIs result from infections in other parts of the body that spread to the bloodstream. Catheter-related BSIs (CRBSIs) occur when an indwelling catheter becomes contaminated, leading to BSI. Only unique BSI episodes were included; thus, episodes for the same patient were included only if the subsequent BSI was caused by a different microorganism > 24 h after the previous BSI, or > 14 days after the previous BSI diagnosis if caused by the same microorganism. The causative pathogens were stratified into the following microbiological categories: *S. aureus*, CONS, enterococci, enterobacterales, non-fermenting Gram-negative bacteria (GNB), fungi, and others. Denominator data for prevalent number of patients on HD at the beginning of each year and total number of regular ambulatory care HD sessions per year were retrieved from HD unit annual reports, and number of patient HD months were calculated per year. Aggregate-level data on patients undergoing maintenance HD at HUG for the years 2014-23 were retrieved from the Swiss renal registry and quality assessment program (SRRQAP) [18].

### Ethical approval

The data for both BSI surveillance and HD registry databases are collected as part of institutional quality improvement programs; thus, institutional review board approval was not required.

### Statistical analysis

BSI incidence density rates were calculated as number of BSI episodes per 100 patient-HD-months and were reported for 3-year periods (2006-08, 2009-11, 2012-14, 2015-17, 2018-20, 2021-23) to account for data sparsity. To assess secular trends, time-period segmented Poisson regression models were applied to 3-yearly aggregated data, using patient-HD-months as an offset. Incidence rate ratios (IRRs) with 95% confidence interval (CI) were presented in relation to the first study period (2006-08). True incidence rates and model-predicted rates were plotted. Overdispersion was evaluated for each modelled stratum using Cameron and Trivedi’s dispersion test (dispersiontest in the AER package) [19], and if present, negative binominal regression models were used.

For the data analysis, if not otherwise stated, we excluded contamination episodes and focused on true monomicrobial BSI episodes. A sensitivity analysis was completed evaluating BSI trends including both monomicrobial and polymicrobial BSIs, with causative pathogens classified according to the following hierarchy (by descending order, *S. aureus*, enterococci, enterobacterales, non-fermenting Gram-negatives, CONS and others). BSI types (primary catheter-related, primary non-catheter related and secondary BSIs), and sources of secondary BSIs were explored. Additionally, separate subgroup trend analyses were performed: (1) to assess trends of MDROs such as MRSA, vancomycin resistant enterococci (VRE) and extended-spectrum beta-lactamase and carbapenemase producing Gram-negative bacteria (MDR-enterobacterales); and (2) to evaluate *S. aureus* rates by BSI type. An explorative analysis was conducted focusing on patients’ characteristics and proportion of patients with catheter HD access for the years 2014-23 based on SRRQAP data. All statistical analyses were carried out using the statistical program ‘R’ version 4.4.2 [20].

## Results

### General description of BSI episodes

**Fig. 1:**
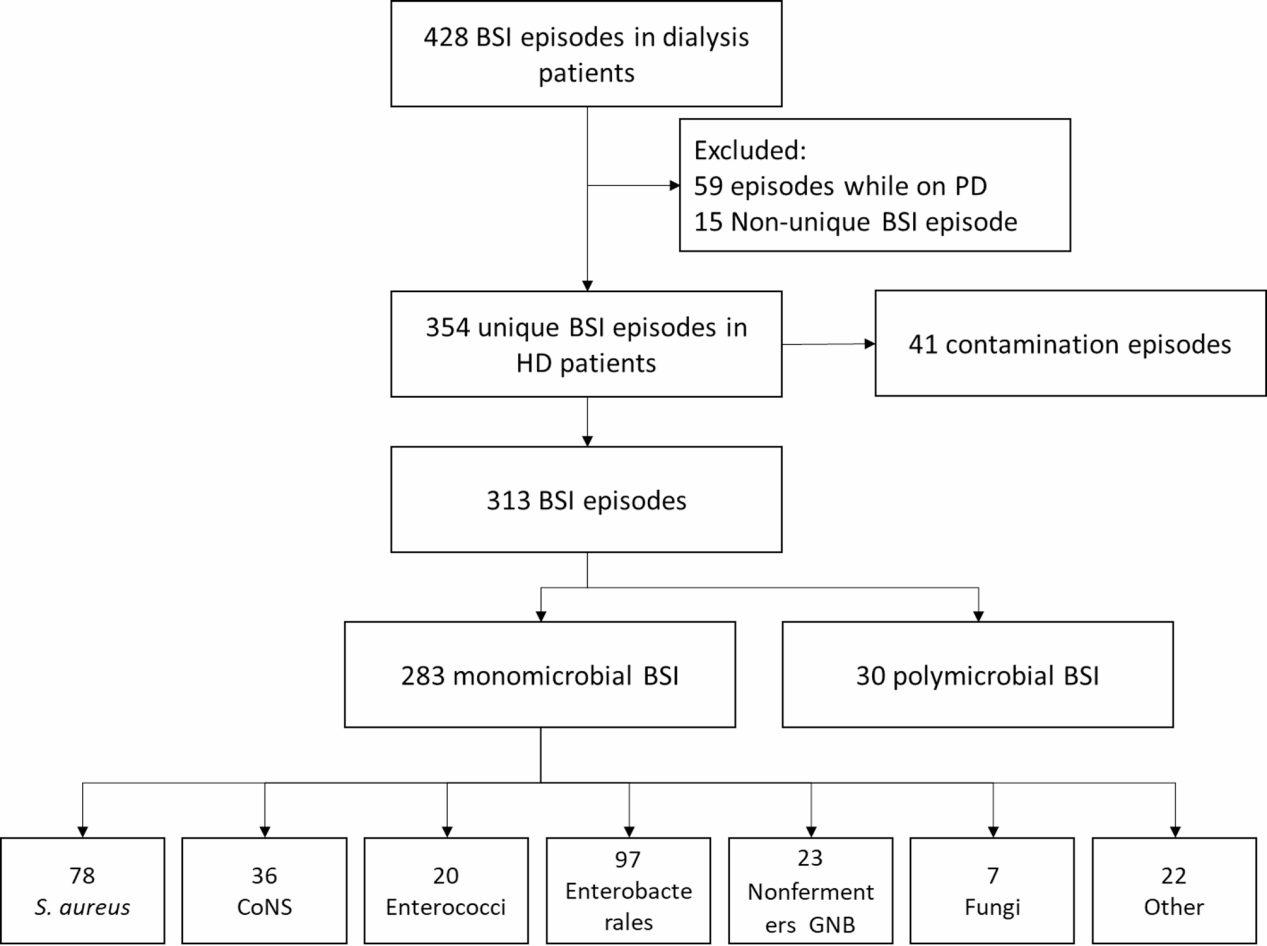
Study flow diagram

During the years 2006-23, a total of 354 unique BSI episodes were detected among 218 HD patients; with 313 true BSIs and 41 contamination episodes (Fig. 1). For 142 patients (65.1%) a single BSI episode was documented; 76 patients (34.9%) experienced more than one unique BSI episode. The mean age of HD patients at the time of BSI diagnosis was 64 ± 15 years and 30% were female. On average, 56 patients (± 12) patients underwent maintenance HD at HUG each year, contributing to a total of 142,983 HD sessions and 11,413 patient-HD-months. Aggregate-level data on HD patients’ characteristics is provided in the supplement (Additional file, Figures S1, S2).

Of all true monomicrobial and polymicrobial BSI episodes (*n* = 313), primary BSIs were most common (64.2%, *n* = 201), including 130 catheter-related and 71 non-catheter related episodes. There were 112 secondary BSIs (35.8%); sources of secondary BSI were urinary tract (*n* = 34, 30.4%), surgical site (*n* = 29, 17.0%), skin and soft tissue (*n* = 14, 12.5%), bone and joint (*n* = 14, 12.5%), pulmonary (*n* = 13, 11.6%), cardiovascular (*n* = 8, 7.1%) and gastrointestinal infections (*n* = 7, 6.3%). The overall BSI rate was 2.7 BSI episodes per 100 patient-HD-months excluding contaminations. Contamination was detected in 11.6% of all BSI episodes corresponding to a contamination rate of 0.38 per 100 patient-HD-months (Table 1).

**Table 1:** Incidence density rates of true monomicrobial bloodstream infection among hemodialysis patients per 100 patient-HD-months by pathogen category and study period, Geneva University Hospitals (2006-23)

Period	Any pathogen (polymicrobial) (*n* = 313)	Monomicrobial (*n* = 284)								Contamination (*n* = 41)
		Any pathogen	Staphylococcus aureus	CoNS	Enterobacterales	Enterococci	Fungi	Non fermenting GNB	Other	
2006-08	3.37	3.28	0.75	0.40	1.02	0.36	0.09	0.49	0.18	0.22
2009-11	3.19	2.77	0.98	0.38	0.70	0.14	0.05	0.28	0.23	0.75
2012-14	3.05	2.85	0.81	0.41	1.02	0.14	0.14	0.20	0.14	0.34
2015-17	2.28	2.06	0.63	0.26	0.95	0.05	0.00	0.00	0.16	0.21
2018-20	2.16	1.94	0.59	0.22	0.59	0.11	0.00	0.11	0.32	0.16
2021-23	2.27	1.83	0.28	0.22	0.83	0.22	0.11	0.06	0.11	0.55
**Overall**	**2.74**	**2.48**	**0.68**	**0.32**	**0.85**	**0.18**	**0.06**	**0.20**	**0.19**	**0.38**

GNB – Gram negative bacteria; CoNS - Coagulase-negative staphylococci

### BSI trends

**Fig. 2:**
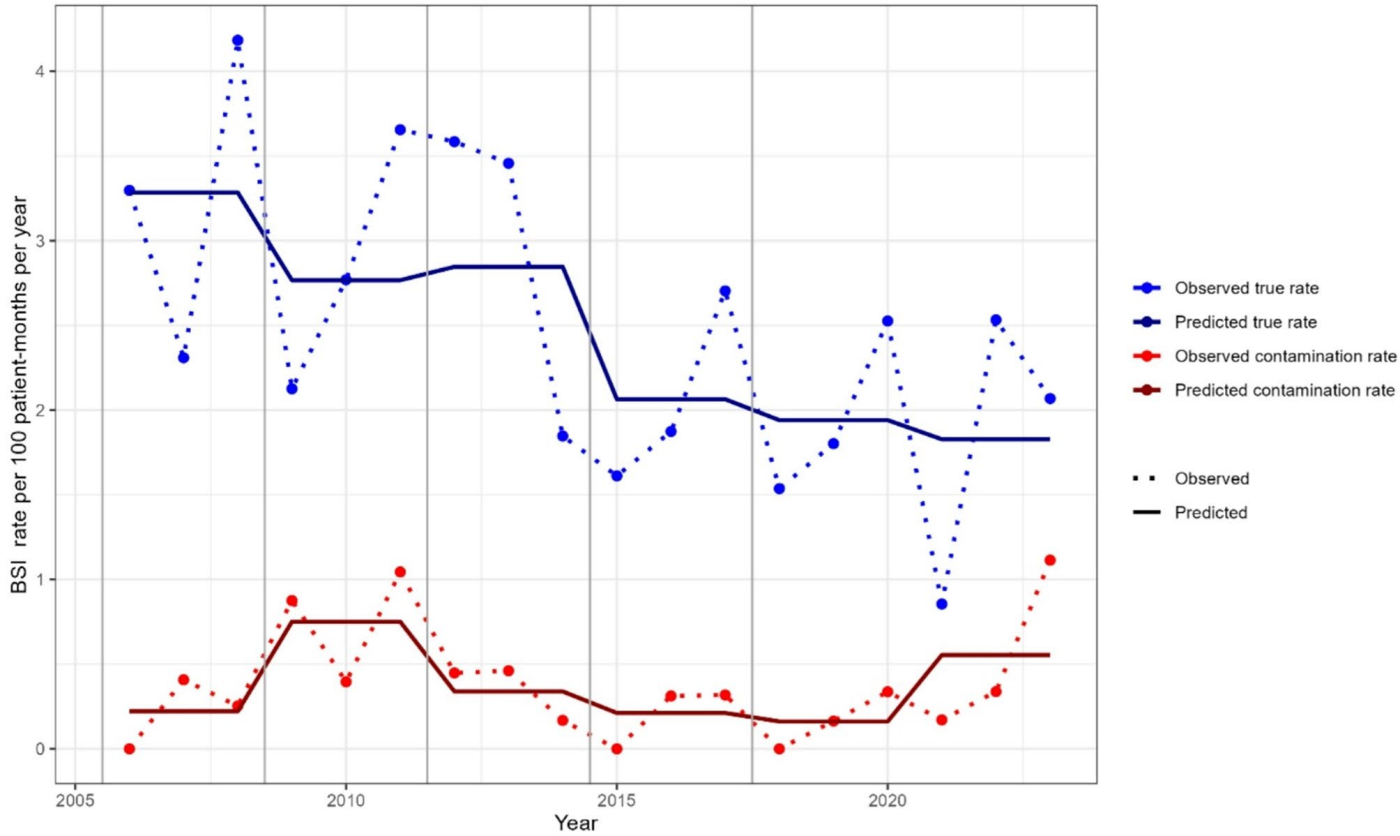
Observed and predicted BSI contamination rates and true BSI incidence per 100 patient-HD-months, Geneva University Hospitals (2003–2021). Lines = predicted rates; dots = observed rates

True BSI rates steadily decreased throughout the study period (Fig. 2). Contamination rates initially increased and then followed a downward trend. A more recent slight increase in contamination rates was observed during the latest period (2021-23), consistent with the COVID-19 pandemic (Fig. 2).

**Fig. 3:**
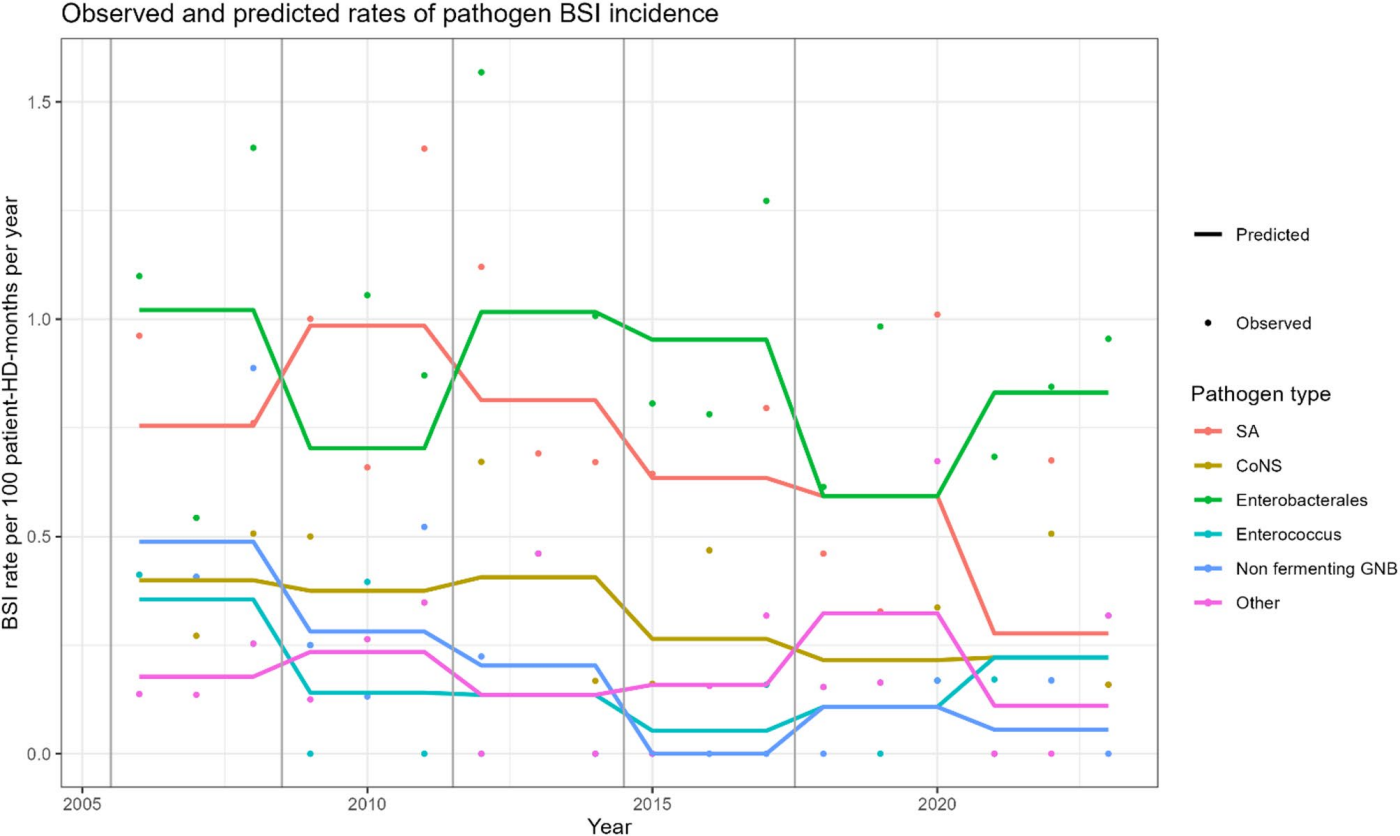
Observed and predicted BSI rates of pathogen specific healthcare-related BSI incidence rates per 100 patient-HD-months, Geneva University Hospitals (2006–2023). Lines = predicted rates; dots = observed rates

Among all true BSIs, 283 episodes were monomicrobial (90.4%). For those, incidence rates over time ranged between 1.8 and 3.3 BSI episodes per 100 patient-HD-months for 2021-23 and 2006-08, respectively (Table 1). Compared to the baseline period of 2006-08, rates of monomicrobial BSI due to any pathogen decreased consistently during the study period reaching a statistically significant reduction starting in 2015-17 compared to the baseline period (2006-08) (Table 2). When looking at pathogen-specific trends, *S. aureus* BSIs increased over the first time period (2009-11) compared to baseline and then followed a steadily decreasing trend. *S. aureus* BSI rates significantly declined by 63% until the latest period compared to baseline (IRR 0.37, 95%CI 0.12–0.93). A decreasing trend was also observed for CONS BSIs, and non-fermenting GNB BSIs, although numbers were small. Conversely, a stable trend in enterobacterales BSI rates was observed. A small number of enterococcal BSI occurred during the study period, with mixed trends (Table 2; Fig. 3). Similar trends for specific pathogens were found in sensitivity analyses including both monomicrobial and polymicrobial BSI episodes (Additional file, Table S1). The pathogen distribution of all BSI episodes is provided in the supplement (Additional file, Figure S3).

**Table 2:** Incidence rate ratios of monomicrobial bacterial bloodstream infection in hemodialysis patients compared to baseline period, overall and per pathogen group, Geneva University Hospitals (2006–23)

	Incidence rate ratio (95% confidence interval)							
BSI causative species/ study period	All pathogens (283)	S. aureus (78)	CoNS (36)*	Enterococci (20)	Enterobacterales (97)	Non fermenting GNB (23)	Others (22)	Contamination (41)
2006-08	1.00	1.00	1.00	1.00	1.00	1.00	1.00	1.00
2009-11	0.84 (0.60–1.18)	1.31 (0.69–2.51)	0.94 (0.35–2.46)	0.40 (0.09–1.37)	0.69 (0.35–1.31)	0.58 (0.20–1.51)	1.32 (0.35–5.34)	**3.38 (1.32–10.34)**
2012-14	0.87 (0.59–1.26)	1.08 (0.50–2.24)	1.02 (0.34–2.82)	0.38 (0.06–1.52)	1.00 (0.51–1.89)	0.42 (0.09–1.33)	0.76 (0.11–3.91)	1.53 (0.42–5.49)
2015-17	**0.63 (0.42–0.92)**	0.84 (0.39–1.75)	0.66 (0.20–1.92)	**0.15 (0.01–0.81)**	0.93 (0.50–1.72)	**	0.89 (0.18–4.06)	0.95 (0.24–3.60)
2018-20	**0.59 (0.39–0.87)**	0.79 (0.36–1.66)	0.54 (0.15–1.66)	0.30 (0.05–1.21)	0.58 (0.27–1.16)	**0.22 (0.03–0.82)**	1.82 (0.52–7.13)	0.73 (0.15–2.97)
2021-23	**0.56 (0.36–0.83)**	**0.37 (0.12–0.93)**	0.55 (0.15–1.70)	0.62 (0.17–1.98)	0.81 (0.42–1.55)	**0.11 (0.01–0.58)**	0.62 (0.09–3.20)	2.50 (0.89–8.01)

In brackets, total number of episodes; **bold** numbers highlight statistical significance (*p* < 0.05)

* Negative binominal model used due to over-dispersion

** no episodes occurred in this period

GNB – Gram-negative bacteria; BSI – bloodstream infection; CoNS - Coagulase-negative staphylococci

### Resistance trends

**Fig. 4:**
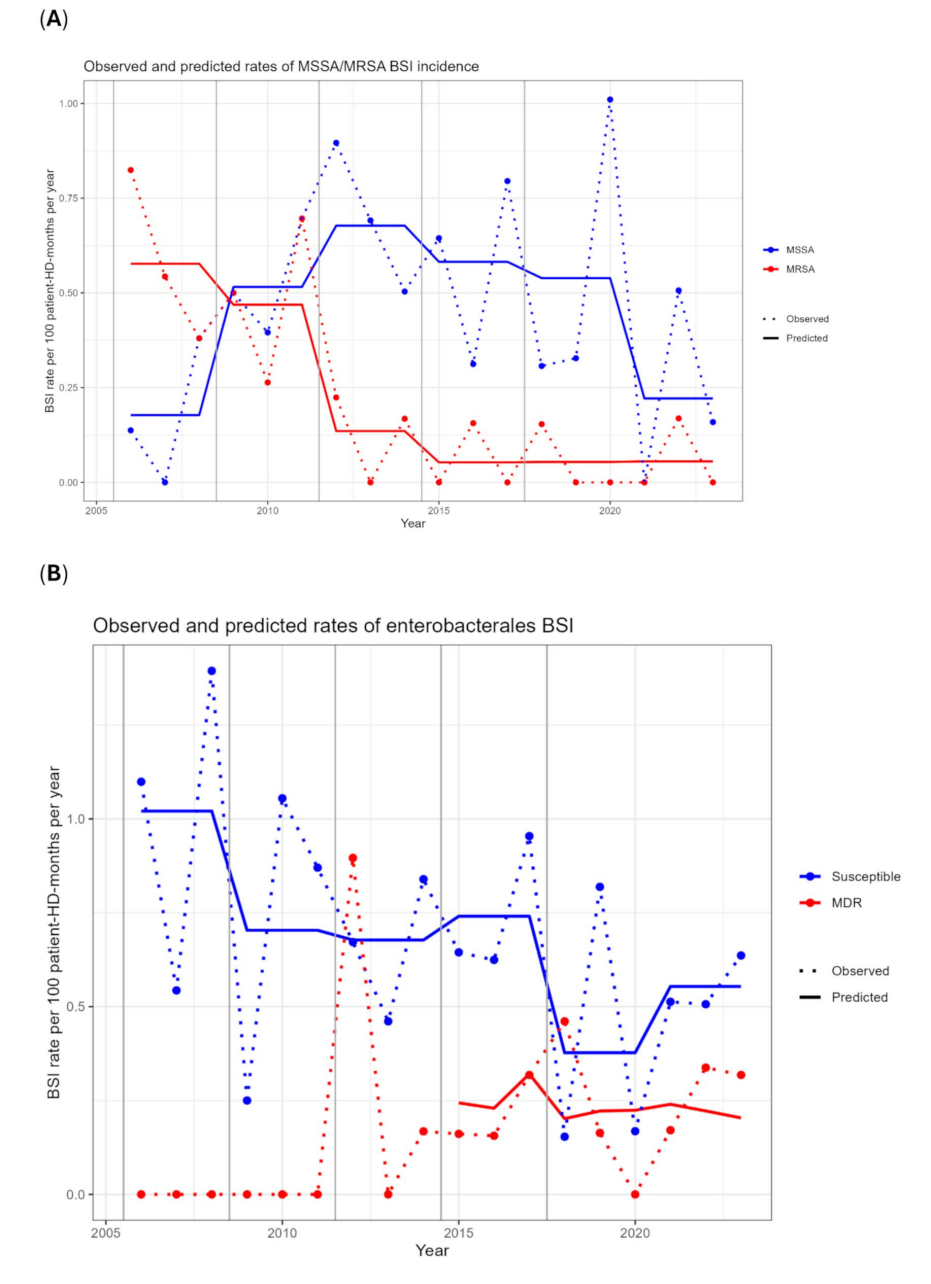
Trends of multidrug resistant and susceptible BSI incidence per 100patient-HD-months in Geneva University Hospitals (2006-2023) for: (**A**) S. aureus (MSSA, MRSA) and (**B**) enterobacterales (susceptible and multidrug resistant). Lines = predicted rates; dots = observed rates

Overall, 47 BSI episodes in HD patients were due to MDROs (28 MRSA, 18 MDR-Enterobacterales, 1 VRE); no multi-resistant non-fermenting Gram-negative BSI was detected (Additional file, Table S2). MRSA BSI rates decreased over time, with a concurrent increase in MSSA BSI until 2012, followed by a plateau; after 2020, MSSA BSIs also decreased substantially (Fig. 4A). For enterobacterales BSIs, multi-resistant strains were first detected in 2012 and remained sporadic in the following years; susceptible enterobacterales showed a slowly decreasing trend as well (Fig. 4B).

### Sources of BSI

When considering all true monomicrobial and polymicrobial BSI episodes, catheter-related BSIs had the highest incidence, corresponding to a rate of 1.1 episodes per 100 patient-HD-months (Additional file, Table S3). As for temporal trends by BSI type, catheter-related BSIs steadily decreased over the study period, reaching a significant reduction from 2015 onwards, and a maximum reduction of 76% in the latest study period (IRR 0.24, 95%CI 0.11–0.49) compared to baseline. Conversely, primary non-catheter related BSIs increased, with a statistically significant increment starting in 2018; secondary BSIs showed an inconsistent trend (Additional file, Table S4 and Figure S4). In a subgroup analysis of monomicrobial *S. aureus* BSIs (*n* = 78), catheter-related BSIs were most prevalent (*n* = 59, 75.6%), and their rate steadily decreased, with a statistically significant reduction in the latest time-period, whereas secondary *S. aureus* BSIs did not show a clear trend (Additional file Table S4, Figure S5).

## Discussion

This study provides unique insights into the secular trends of HD-associated BSI in the same institution over 18 years. A consistent decreasing trend was observed in overall BSI rates compared to the baseline period (2006-08), with a statistically significant reduction from 2015 onwards. This finding confirms the preventable nature of this healthcare-related infection in vulnerable HD patients, and the potentially avertable burden for healthcare systems. The observed reduction in BSI rates was mainly driven by a decrease in *S. aureus* BSIs, which might be associated, in part, with the intervention of systematic *S. aureus* screening and targeted decolonization of carriers set in place since 2000 [11]. While *S. aureus* BSI rates decreased, BSIs due to enterobacterales remained stable, suggesting no replacement of *S. aureus* BSIs by other pathogens. Temporal trends of BSI due to specific pathogens were not assessed in previous studies. A nation-wide study in Denmark evaluated temporal trends of sepsis in HD patients that were found stable (1996–2017), except for a significant increase in the latest study period for which no apparent reason was found (for 2014-17 compared to baseline 1996–2000) [21].

Of note, MDRO infection prevention is of utmost importance in HD patients due to their constant contact with healthcare and higher burden due to resistance [22, 23]. In our study, MDRO BSIs were rare events and followed a similar decreasing trend. MRSA BSI rates declined promptly following the baseline period; multi-drug resistant enterobacterales BSIs were first detected in 2012 and their incidence was sporadic thereafter.

An overall BSI rate of 2.7 BSI episodes per 100 patient-HD-months was found in our study. This is notably higher than the rates reported in the 2014 National Healthcare Safety Network (NHSN) Dialysis Event Surveillance Report, that documented an overall BSI rate of 0.64 BSIs per 100 patient-HD-months, and 2.2 BSIs per 100 patient-HD-months for patients carrying a central venous catheter [24]. The higher incidence rates observed in our study could be related to differences in patient case-mix or in HD-access types with higher proportions of vascular catheter HD-access. Also, differences in surveillance methods could have had an effect; our approach included all BSI episodes in HD patients, regardless of the timing or setting of blood culture collection or their attribution to HD status, in contrast, the NHSN report only captured blood cultures collected in outpatient dialysis settings or within the first day of hospitalization following a dialysis session.

Catheter-related BSIs were most common in our HD patients and were responsible for two thirds of all BSI episodes, with incidence rates as high as 1.8 episodes per 100 patient-HD-months at the years 2006-11, that decreased to 0.4 episodes per 100 patient-HD-months in the latest study period. From 2012 onwards, a significant decrease in primary catheter-related BSI rates was observed, but not in primary non-catheter related BSIs or secondary BSIs. It is well known that HD catheters are the main driving force for developing catheter-related BSI in HD patients [12, 25]. Central venous catheters for HD access were previously associated with the highest risk for HD-associated BSI compared to the use of fistula or graft [9]. Nowadays, catheter avoidance is considered a valuable approach aiming to reduce BSI in HD patients, yet catheter use may still be inevitable in specific patient groups. In our institution, catheters were used for HD-access for almost half of the patients in the years 2014-23, with a non-significant decrease in proportions of catheter use in the later study years; this trend might have contributed to the decline in catheter-related BSIs, but data on the distribution of HD-access type was not consistently available for the entire study period. Other preventive measures such as catheter exit site ointments and dressings, catheter hub devices and antimicrobial lock solutions can be applied to reduce BSI rates relying on variable degrees of evidence [26, 27]. Specifically for *S. aureus* BSIs, most episodes were catheter-related primary BSIs, and these decreased over time. This might hint at a beneficial effect of systematic *S. aureus* screening and targeted decolonization of carrier HD patients [11]. However, other factors, such as changes in catheter care practices and patient characteristics, and hospital-wide infection prevention and control measures may have also contributed to these trends. Specifically, horizontal interventions such as hand hygiene monitoring and surveillance of hospital-acquired infections were implemented in our hospital during the entire study period. Furthermore, new staff working in hemodialysis receives in-depth education on catheter care, other than that no specific intervention was implemented.

Of note, a substantial prevention potential of HD-associated BSIs was observed in previous studies [10]. In a large cluster-randomized trial involving patients in 211 HD facility matched pairs in North America, improved CVC site care resulted in 20% reduction in BSI rates in intervention facilities compared to control with post-intervention BSI rates of 0.81 versus 1.04 per 1,000 CVC-days [28]. A recent cluster-randomized trial in German HD outpatient units, showed that implementation of a multimodal infection prevention strategy including infection surveillance, hand hygiene observation with feedback, training on aseptic procedures, and patient education resulted in 54% decrease in dialysis-associated infection events including BSIs, local access-related infections and intravenous antibiotic starts [29].

While our study portrays findings from a comprehensive BSI surveillance effort for HD patients, we recognize some limitations. First, as data collection was done for surveillance purposes, important clinical features of HD patients were not available. Secondly, assessment of specific HD-catheter-related BSIs was not possible, as the specific catheter type was not consistently reported. Yet, it is reasonable to assume that most catheter-related BSIs in HD patients were associated with the catheter used for HD-access. Third, surveillance methods were changed in 2016, after introduction of ECDC protocols. While we do not expect a major bias in the overall BSI rates, source attribution of BSI episodes—particularly catheter-related BSIs—may have been affected. Under the CDC definitions, classification of catheter-related BSI typically required microbiological confirmation (catheter tip culture or differential time to positivity), whereas the ECDC definitions rely more on clinical criteria without requiring microbiological evidence from the catheter. This shift may have led to a relative increase in the number of BSI episodes attributed to central lines after 2016, supporting the interpretation of a true decrease in catheter-related BSI rates over time. Lastly, as a single center study from a high-income setting, our findings might not be generalizable to other settings, with different health care systems and hyperendemic MDROs.

## Conclusions

Primary BSIs, and catheter-related BSIs in particular, dominate BSI patterns in HD patients. Pathogen-specific BSI trends revealed a significant reduction in *S. aureus* BSIs, without replacement by other pathogens. Our findings highlight the importance of infection prevention in HD. Further studies to examine the effectiveness and sustainability of different infection prevention interventions in this population are warranted.

## Supplementary Information

Below is the link to the electronic supplementary material.

Supplementary Material 1

## Data Availability

No datasets were generated or analysed during the current study.
