# Supplementary material for: Secular trends of bloodstream infections in hemodialysis patients: insights from a longitudinal Swiss study
Source: Antimicrob Resist Infect Control. 2025 Aug 20;14:100. doi: 10.1186/s13756-025-01620-8 (PMC12366405; doi:10.1186/s13756-025-01620-8)

**Additional file – supplementary material:**

**Secular trends of bloodstream infections in hemodialysis patients: insights from a longitudinal Swiss study**

Table S1 Sensitivity trend analysis including monomicrobial and polymicrobial bacterial BSI in HD patients*

|  | **Incidence rate ratio (95% confidence interval)** | | | | | | |  |
| --- | --- | --- | --- | --- | --- | --- | --- | --- |
| **BSI causative species/**  **study period** | **All pathogens (313)** | **S. aureus (83)** | **CONS (36)** | **Enterococci (29)** | **Enterobacterales (106)** | **Non-fermenters GN (27)** | **Other (25)** | |
| 2006-08 | 1.00 | 1.00 | 1.00 | 1.00 | 1.00 | 1.00 | 1.00 | |
| 2009-11 | 0.95 (0.68-1.31) | 1.35 (0.73-2.53) | 0.94 (0.35-2.46) | 0.47 (0.13-1.44) | 0.92 (0.50-1.67) | 0.58 (0.20-1.51) | 1.58 (0.45-6.20) | |
| 2012-14 | 0.90 (0.62-1.30) | 1.02 (0.48-2.09) | 1.02 (0.34-2.82) | 0.85 (0.26-2.45) | 1.00 (0.51-1.89) | 0.42 (0.09-1.33) | 0.76 (0.11-3.91) | |
| 2015-17 | **0.67 (0.46-0.98)** | 0.79 (0.37-1.63) | 0.66 (0.20-1.92) | **0.13 (0.01-0.70)** | 0.98 (0.53-1.81) | **0.22 (0.03-0.81)** | 1.19 (0.25-5.04) | |
| 2018-20 | **0.64 (0.43-0.93)** | 0.74 (0.34-1.55) | 0.54 (0.15-1.66) | 0.54 (0.15-1.66) | 0.63 (0.31-1.25) | **0.22 (0.03-0.82)** | 2.13 (0.64-8.11) | |
| 2021-23 | **0.67 (0.46-0.98)** | 0.49 (0.19-1.11) | 0.55 (0.15-1.70) | 0.83 (0.28-2.31) | 0.92 (0.49-1.72) | 0.34 (0.08-1.09) | 0.62 (0.09-3.20) | |

In brackets, total number of episodes; Bold resembles statistical significance (p<0.05); GN – gram negatives.

*Causative pathogens were classified according to the following hierarchy (by descending order, *S. aureus*, enterococci, enterobacterales, non-fermenting Gram negatives, CONS, fungi and others).

Table S2 Susceptible versus resistant BSI trends in HD patients*

|  | **Incidence rate ratio (95% confidence interval)** | | | |
| --- | --- | --- | --- | --- |
| **BSI causative species/**  **study period** | **MRSA (28)** | **MSSA (50)** | **MDR Enterobacterales (18)**** | **Susceptible Enterobacterales (79)** |
| 2006-08 | 1.00 | 1.00 | ***- | 1.00 |
| 2009-11 | 0.81 (0.35-1.85) | 2.91 (0.99-10.48) | ***- | 0.69 (0.35-1.31) |
| 2012-14 | **0.23 (0.04-0.85)** | **3.85 (1.25-13.91)** | 1.00 | 0.66 (0.30-1.36) |
| 2015-17 | **0.09 (0.01-0.46)** | **3.28 (1.12-11.83)** | 0.62 (0.15-2.36) | 0.73 (0.36-1.39) |
| 2018-20 | **0.09 (0.01-0.47)** | **3.04 (1.02-11.7)** | 0.64 (0.16-2.41) | **0.37 (0.15-0.82)** |
| 2021-23 | **0.10 (0.01-0.48)** | 1.25 (0.30-5.28) | 0.82 (0.23-2.94) | 0.54 (0.25-1.11) |

In brackets, total number of episodes; Bold resembles statistical significance (p<0.05); MDR – multidrug resistant; MSSA – methicillin susceptible *S. aureus*; MRSA – methicillin Resistant *S. aureus.*

* One vancomycin resistant enterococcus BSI episode and no MDR-non-fermenting BSI episodes occurred in HD patients.

** MDR enterobacterales – 16 Extended-spectrum beta-lactamase, and 2 carbapenemase-producing isoaltes

***no events in this time period.

Table S3 – incidence rates of BSI during the study period by BSI type per 100 patient-HD months

| study period/ BSI type | BJ | CV | GI | OTH | PULM | SSI | SSTI | UTI | CA | NCA |
| --- | --- | --- | --- | --- | --- | --- | --- | --- | --- | --- |
| 2006-08 | 0.27 | 0.13 | 0.13 | 0.00 | 0.09 | 0.18 | 0.09 | 0.36 | 1.82 | 0.31 |
| 2009-11 | 0.14 | 0.00 | 0.05 | 0.00 | 0.09 | 0.05 | 0.19 | 0.33 | 1.78 | 0.56 |
| 2012-14 | 0.14 | 0.14 | 0.00 | 0.00 | 0.27 | 0.20 | 0.14 | 0.34 | 1.29 | 0.54 |
| 2015-17 | 0.00 | 0.11 | 0.11 | 0.11 | 0.11 | 0.26 | 0.00 | 0.21 | 0.79 | 0.58 |
| 2018-20 | 0.16 | 0.05 | 0.00 | 0.00 | 0.05 | 0.05 | 0.27 | 0.16 | 0.49 | 0.92 |
| 2021-23 | 0.00 | 0.00 | 0.06 | 0.06 | 0.11 | 0.28 | 0.06 | 0.39 | 0.44 | 0.89 |
| **Overall** | **0.12** | **0.07** | **0.06** | **0.03** | **0.11** | **0.17** | **0.12** | **0.30** | **1.14** | **0.62** |

BSI – blood stream infection; BJ: bone and joint; CV cardiovascular; GI gastrointestinal; OTH other; PULM pulmonary; SSI surgical site infection; SSTI skin and soft tissue infection; UTI urinary tract infection; CA catheter associated (primary); NCA non-catheter associated (primary).

Table S4 – Trends of BSI classification over the study period – all true BSI episodes

|  | **Incidence rate ratio (95% confidence interval)**  **All pathogens* (313)** | | | **Incidence rate ratio (95% confidence interval)**  **monomicrobial *S. aureus* (73)** | |
| --- | --- | --- | --- | --- | --- |
| **BSI classification/**  **study period** | **Primary non-catheter related BSI (71)** | **Catheter related BSI (130)** | **Secondary BSI (112)** | **Catheter related BSI (59)** | **Secondary BSI (14)** |
| 2006-08 | 1.00 | 1.00 | 1.00 | 1.00 | 1.00 |
| 2009-11 | 1.81 (0.73-4.87) | 0.98 (0.63-1.52) | 0.68 (0.37-1.22) | 1.51 (0.77-3.05) | 0.35 (0.02-2.75) |
| 2012-14 | 1.74 (0.63-4.98) | 0.71 (0.40-1.20) | 0.98 (0.53-1.76) | 0.98 (0.41-2.24) | 1.53 (0.28-8.25) |
| 2015-17 | 1.87 (0.74-5.09) | **0.44 (0.23-0.77)** | 0.72 (0.39-1.31) | 0.77 (0.32-1.75) | 0.40 (0.02-3.10) |
| 2018-20 | **2.95 (1.27-7.63)** | **0.27 (0.12-0.52)** | 0.61 (0.31-1.13) | 0.43 (0.14-1.13) | 1.62 (0.36-8.22) |
| 2021-23 | **2.85 (1.22-7.42)** | **0.24 (0.11-0.49)** | 0.76 (0.41-1.37) | **0.18 (0.03-0.64)** | 0.83 (0.11-5.02) |

In brackets, total number of episodes with classification; Bold resembles statistical significance (p<0.05);

* monomicrobial and poly microbial; Primary non-catheter related BSI due to *S. aureus* occurred in 5 episodes, these were not modelled due to sparsity of events.

Figure S1 Patients characteristics during the years 2014-2023

**D**

During the years 2014-23 significant decrease in mean age and Charlson’s score of our patients were observed (p=0.002 and p=0.009, respectively); and marginally non-significant decrease in prevalent catheter use (p=0.072). On the other hand, no significant difference was observed in gender distribution (p=0.875).

Figure S2 – Overall mortality among HD patients during the years 2014-2023


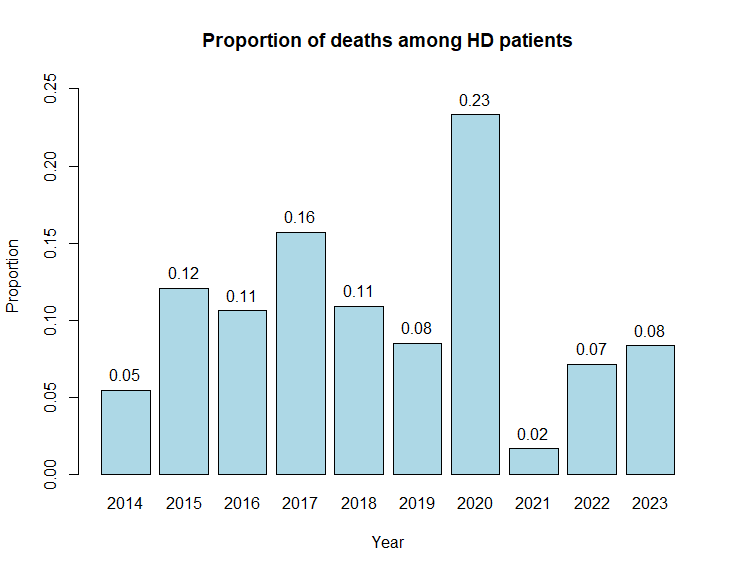


Increased mortality risk was observed in 2020 compared to other years based on annual reporting to the national registry. This finding might explain the decrease in mean age observed in Fig S2.

Figure S3 Distribution of blood isolates in BSI among HD patients including all true BSIs – contamination episodes excluded


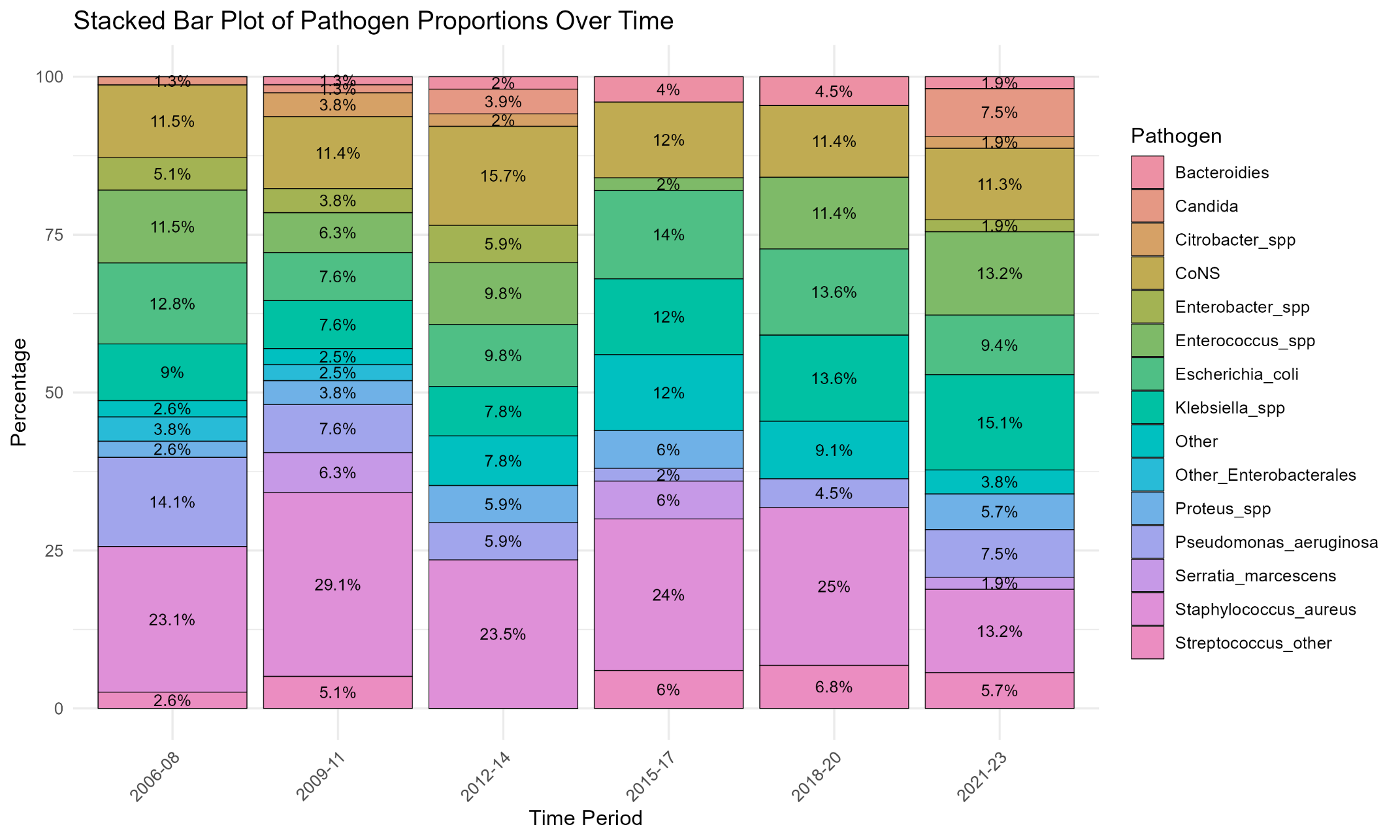


Figure S4 Incidence density of monomicrobial BSIs during the study period by type


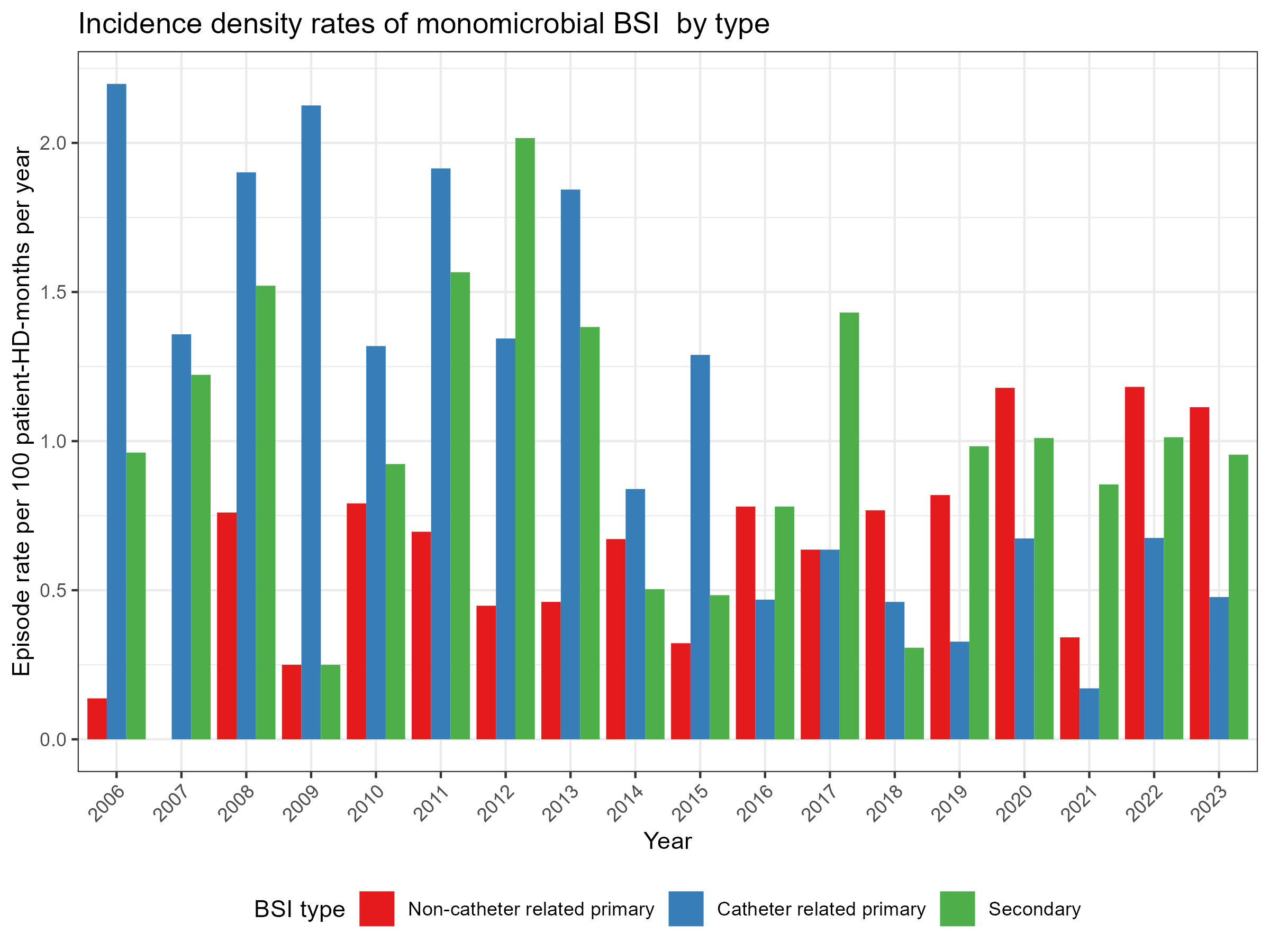


Figure S5 – Incidence density of S. aureus BSIs during the study period by type


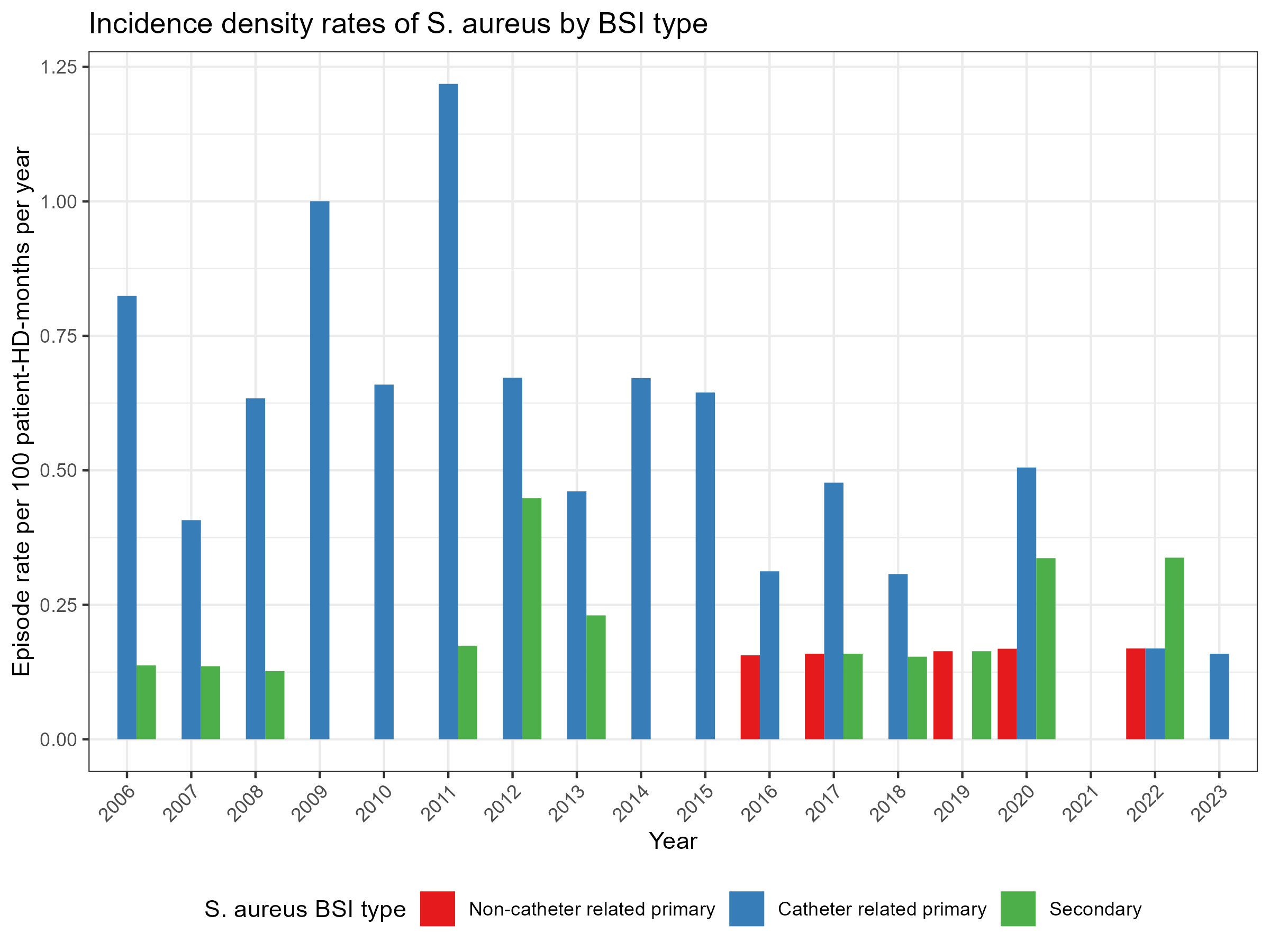

Supplement: Supplementary file 1 — Supplementary Material 1 [file 13756_2025_1620_MOESM1_ESM.docx]
